# Understanding Social Media Strategies to Improve Engagement in and Equitable Dissemination of Online Cancer Nutrition Information: Cross-Sectional Study

**DOI:** 10.2196/69886

**Published:** 2026-08-14

**Authors:** Ellie Harrison, Sara Buzali-Soto, Eileen Rillamas-Sun, Heather Greenlee, Echo L Warner

**Affiliations:** 1 Department of Epidemiology, School of Public Health, University of Washington, Seattle, United States; 2 Fred Hutch Cancer Center, Seattle, United States; 3 Department of Pediatrics, Huntsman Cancer Institute, University of Utah, 2000 Circle of HopeSalt Lake City, UT, 84112, United States, 1-801-244-7040

**Keywords:** Facebook, content analysis, social media, engagement, cancer prevention, nutrition education, bilingual

## Abstract

**Background:**

Social media is a common source of cancer information for patients, caregivers, and survivors. Factors influencing engagement with oncology nutrition content on social media are not well-defined, despite the high prevalence of social media use for health information. The impact of bilingual content strategies on that engagement is also poorly understood.

**Objective:**

This study aims to describe changes in postlevel engagement on a Facebook page used to disseminate evidence-based cancer nutrition information during the period before and after the implementation of a bilingual, multiformat content strategy and to identify creative and linguistic features associated with audience engagement at the post level.

**Methods:**

This cross-sectional study analyzed 306 Facebook posts from Cook for Your Life over 12 months, comprising 9 months before the strategy change (T1) and 3 months after (T2). Postlevel engagement metrics (likes, comments, shares, and link clicks) and aggregated user demographics were obtained from Meta Business Suite. Trained coders annotated visual and creative features, including branding, post shape, and infographics, as well as topical categories. Linguistic Inquiry and Word Count characterized language features. Group differences across time periods were tested using *t* tests and chi-square tests. Generalized linear models estimated associations between post characteristics and engagement, adjusting for the time period.

**Results:**

Users who engaged with oncology nutrition Facebook content (N=10,196) were mostly female (n=9500, 93.2%) participants, over 35 years of age (n=9311, 91.4%), based in the United States (n=6708, 71.4%), and English-language users (n=9419, 93.4%). Engagement tripled from T1 (mean 5.3, SD 4.2) to T2 (mean 17.7, SD 37.9; *P*<.001). Creative features such as branding and format were positively associated with engagement. Linguistically, there was a shift toward longer sentence structures, simplified vocabulary, increased analytical language, and a decline in emotionally charged and cognitive language in posts during T2. In models controlling for time period, posts that used bilingual content had 1.72-fold higher odds of audience engagement than English-only posts (95% CI 1.05‐2.80).

**Conclusions:**

Implementing a bilingual, multiformat content strategy may promote engagement with oncology nutrition information on Facebook and enhance the equitable dissemination of health information. The use of creative visual design elements in combination with language that is more analytical and emotionally neutral was associated with higher engagement with educational public health content. Future research should test bilingual language presentation more directly and examine whether exposure to such content influences health beliefs and downstream behaviors across additional platforms.

## Introduction

### Emerging Health Communication on Social Media

The pervasive use of the internet, driven by the emergence of social media platforms, has revolutionized communication patterns, particularly within health-related contexts [1]. Social media, ranking as the third most frequent online activity, has become an integral part of human interaction, facilitating the exchange of ideas, information sharing, and the creation of online communities [2,3]. With the increasing prevalence of personal wireless devices, the popularity of social media applications continues to grow worldwide [4-6]. This observation has prompted health communication experts to explore innovative opportunities for using social media to enhance population health [3,7-11].

Social media platforms are powerful tools for health communication interventions and offer opportunities to improve outreach and impact [12-14]. Notably, cancer organizations have increasingly embraced social media for outreach and patient support and have promoted technology-based interventions that use social media to support patients and caregivers in the period after a cancer diagnosis [15]. Studies across multiple scientific disciplines have highlighted the potential of social media to improve health through the efficient exchange of user-generated health information, increased perceived social support, and the facilitation of public health programs such as dietary interventions [16-23].

It is valuable to study the language organizations use in online cancer communities related to oncology nutrition education, considering the rapid transformations in the communication landscape brought on by participative internet use and social media alongside the potential for social media to amplify misinformation [24]. Research studying the association between engagement and the language used within social media aligns with the National Cancer Institute’s focus on bringing cancer research studies to individuals in their communities [25]. The first step in this effort is to identify the characteristics of current social media users. *Facebook* is a social media platform that allows users to share and interact with stories, images, and videos. In the United States, Facebook is widely used within the adult population, more commonly by females than males [26]. On this platform, *engagement* is defined as any action users take on a Facebook page or post [27]. The most common examples of Facebook engagement actions are reactions (eg, likes), comments, and shares, but engagement can also include video views, link clicks, or post saves. Understanding social media usage and how people engage with information online will support health communication efforts aiming to use social media effectively to promote the dissemination of trusted, evidence-based oncology nutrition content online. This may inform the work of health science institutions to design effective population health campaigns that use digital technology.

### Cook for Your Life

Cook for Your Life (CFYL), a website based at Fred Hutchinson Cancer Center (FHCC), stands out as a pioneer in promoting healthy cooking for individuals with cancer [28]. Founded in 2007 by a 2-time cancer survivor as a community-based nonprofit in New York City teaching in-person culinary and nutrition courses to cancer survivors, CFYL launched its website in 2012 to address the unmet need for culinary and nutrition education in the field of oncology. In 2019, the website was transferred to FHCC to continue serving as a bilingual (English/Spanish) research platform and community resource under their institutional support. The content produced by CFYL, which is grounded in science and supported by 2 advisory boards composed of FHCC leaders and experts—Scientific & Medical Advisors and Clinical Oncology Nutrition Advisors—caters to patients with cancer, survivors, individuals interested in cancer prevention, as well as health care professionals and oncology societies. Disseminated through social media platforms such as Facebook, X (formerly known as Twitter), Pinterest, YouTube, and Instagram, CFYL’s library comprises a rich collection of science-based recipes, articles, and videos.

In a strategic move in September 2021, CFYL implemented a bilingual multiformat social media strategy, producing content in both English and Spanish. This targeted approach, grounded in evidence-based communication techniques, aimed to enhance engagement and broaden the audience for oncology nutrition content. This strategy was executed on the CFYL Facebook page, and the content produced from the strategy is publicly available for review online. The evidence for implementing this content strategy drew on visual communication and health communication research that links visuals and branding to higher social sharing, greater engagement, and improved persuasive impact for health information. The incorporation of bilingual content into CFYL’s social media strategy serves as a unique opportunity to investigate linguistic changes in Facebook content and their influence on engagement.

With that being said, the importance of using social media to communicate science-based information to people affected by cancer warrants further research on this topic, and few studies have examined which bilingual, visual, and linguistic features of oncology nutrition Facebook posts are associated with engagement. We hope to unravel the characteristics of social media posts that promote engagement with the CFYL content on Facebook, focusing on nutrition and cancer, before and after integrating targeted bilingual, multiformat content into their social media approach. Our objective is to describe changes in postlevel engagement on the CFYL Facebook page in the period before and after the implementation of this content strategy and to identify creative and linguistic features associated with audience engagement at the post level. We aim to deepen our understanding of the factors influencing user engagement with health information via social media platforms, specifically within online cancer communities.

## Methods

### Study Timeline

This descriptive cross-sectional study uses linguistic content analysis to examine textual data from Facebook posts about nutrition and cancer prevention and to describe (1) indicators of social engagement, (2) valence (ie, positive/negative terms), and (3) linguistic content (eg, emotional terms/pronouns). The study analyzes CFYL Facebook content over a 12-month period during the COVID-19 pandemic. We divided the 12-month observation window into 2 prespecified periods based on the implementation of a revised social media strategy in September 2021. Time point 1 (T1) represented 9 months before implementation (January 1-September 30, 2021; n=202 posts), and time point 2 (T2) represented the first 3 months after implementation (October 1-December 31, 2021; n=104 posts). T2 was intentionally restricted to the immediate postimplementation period to capture early changes in posting practices while minimizing contamination from later programmatic changes or cultural events. This temporal division facilitated the examination of post characteristics associated with engagement, linguistic content, and other factors in the introduction of a bilingual, multiformat social media strategy by CFYL.

### Facebook Metadata and Sampling

The analytic sample comprised 306 original posts published by the official CFYL Facebook page during the 12-month observation period from January 1, 2021, through December 31, 2021. Each post was created and published by CFYL page administrators as part of the organization’s dissemination of evidence-based oncology nutrition content, including links to CFYL recipes, articles, videos, and branded educational graphics. The unit of analysis was the individual CFYL-authored Facebook post. User-generated comments and posts from non-CFYL accounts were not included as analytic units; engagement with CFYL posts was captured through platform-generated metadata.

Postlevel reach, impressions, engagement, and other key metrics (post type: photo, link, video, or albums) were exported for each CFYL-authored post from Meta Business Suite, the standard analytics interface for Facebook pages. Reach reflects the number of unique accounts that saw a post; impressions reflect total on-screen displays, and engagement aggregates user interactions such as reactions/likes, comments, shares, and link clicks. These are widely used reporting metrics and serve as a data source for digital health research. Meta Business Suite also provided aggregate demographic information describing the audience that interacted with the CFYL Facebook page. Demographic variables extracted from Meta Business Suite include gender, age group, language spoken, and country. Access to this Facebook Metadata is limited to the administrators of the CFYL Facebook account and is not publicly available.

### Qualitative Content Analysis

Certain variables of interest were not available directly from Meta Business Suite (branding, post shape, image content, and topic). Content codes were manually collected from the CFYL Facebook page by a research assistant. This manual extraction included the post text in both English and Spanish, the number of comments and emojis, and image content data. Each post was assigned a unique identifier to enable linkage to Meta Business Suite variables. Additional data included postpublication dates and hyperlinks. These manual content codes were used to construct independent variables for analysis to contextualize observed pre-post shifts in creative strategy and topical focus. To ensure data accuracy, another investigator quality-checked this manually collected data. Next, to analyze how the content of posts was associated with engagement, we performed qualitative content analysis. A codebook from prior social media research was adapted and implemented to code the visual and topical content of posts [29]. Discrepancies and inconsistencies were discussed among the research team, and the codebook was refined over time (Multimedia Appendix 1). User data were grouped according to categorical coding units with adequate variation for statistical comparisons. Manual content codes (branding, post shape, and topic) were used to construct independent variables for quantitative analyses and to contextualize observed pre-post shifts in creative strategy and topical focus.

### Linguistic Variables

To analyze the linguistic content of CFYL Facebook posts, Linguistic Inquiry and Word Count (LIWC) was used. LIWC is a widely recognized content analysis platform that provides valuable insights into the linguistic characteristics of textual content [30]. It includes various dimensions such as emotional tone, positivity, negativity, pronouns, and numerous other linguistic attributes. The selection of specific LIWC dimensions was based on the research questions, hypotheses, and preliminary findings, aligning with the study’s objectives. LIWC’s “Analytic” summary dimension reflects formal, logical, and hierarchical thinking styles, with higher values indicating more analytic language. “Tone” summarizes overall emotional tone, and “Big Words” reflects the use of longer words (commonly operationalized as words with 6 or more letters).

There are also more specific index variables used to assess distinct linguistic processes. For example, the “Cognitive” dimension indicates reasoning and stance-taking, “Authentic” indicates the use of a more personal, self-disclosing, first-person style, and “Perceptual” notes the presence of sensory language. Informal language indicators such as “Conversational” and “Netspeak” capture a more chat-like style and internet shorthand. Social orientation is captured by “Social processes” and related subcategories (eg, “Social referents,” “Family,” and gender references), whereas “Affect,” “Emotion,” “Positive emotion,” and “Negative emotion” quantify the proportion of emotion-laden words. Topic-oriented categories such as “Health” and “Illness” index the extent to which posts reference health and disease experiences. The “Netspeak” dimension describes internet-specific language such as emojis and internet acronyms (eg, LOL [laughing out loud] and OMG [oh my god]).

A stop list was applied to exclude noncontent words, ensuring that the analysis focused on meaningful language. Textual data from hashtags were also omitted from the analysis, as they were considered irrelevant and repetitive content. Then, LIWC was applied to estimate the percentage of terms in each post containing different language types (with scores ranging from 0 to 100). Higher values indicate a greater proportion of words in the post belonging to that category (or higher scores on LIWC summary dimensions), and lower values indicate less frequent use of that linguistic feature.

### Engagement Outcomes

Our primary outcome variables were indicators of social engagement within online cancer communities, defined as the number of likes, comments, and shares on each CFYL Facebook post. This definition using postlevel engagement was selected based on its prior use in digital health research and the presence of these features across other social media platforms [23,31]. We also extracted postlevel engaged user metrics from Meta Business Suite, which reflect the number of users who performed one or more engagement actions on the post. Outcomes of engagement included postlevel summary statistics (mean, SD) for each time period. High engagement indicated that posts were relevant to the target audience. Reach and impressions were also extracted to characterize post exposure. We integrated platform analytics (Meta Business Suite), manual content coding of post features and topics, and LIWC text analysis to characterize how changes in post design, topic focus, and linguistic style aligned with changes in engagement across time periods.

### Statistical Analysis

To facilitate statistical comparisons, textual data were grouped based on categorical coding units with sufficient variation. We descriptively summarized post characteristics, engagement metrics, and aggregate audience demographics obtained from Meta Business Suite using both total counts and postlevel distributional summaries. Bivariate analyses were used to compare postlevel characteristics between the preimplementation (T1) and postimplementation (T2) periods. Independent-samples *t* tests were used for continuous variables, including engagement metrics and LIWC summary measures, whereas chi-square tests were used for categorical variables such as post format, branding, bilingual presentation, and topic categories. To examine associations between post characteristics and engagement while accounting for differences between time periods, we fit generalized linear models, treating the number of total engaged users as a continuous postlevel outcome, and reported odds ratios (ORs) with 95% CIs. In these models, the time period was included as a covariate to account for temporal differences between T1 and T2. All statistical tests were 2-sided, and *P*<.05 was considered statistically significant. This regression model provides insights into the dynamics of engagement and linguistic content on the CFYL Facebook page while controlling for temporality.

### Ethical Considerations

This study analyzed CFYL’s public Facebook posts and aggregated, deidentified engagement metrics exported from Meta Business Suite at the post level. Manual coding was limited to CFYL-owned post text and images, not user comments or profiles. No direct identifiers (eg, names, usernames, and profile links) were collected, and no individual user content from private accounts or direct messages was accessed. Analyses were conducted on deidentified, aggregated data; demographic information (eg, age group, country, and language) was available only in platform-provided summary form. The FHCC determined the project exempt from institutional review board approval, and no individual consent was required. FHCC provides funding for CFYL and supported research activities. Procedures for data use complied with ethical expectations for internet-based research and did not involve automated scraping or circumvention of platform protections.

## Results

### Characteristics of Individuals Who Engaged With CFYL Content on Facebook

The final analytic dataset included 306 original CFYL-authored Facebook posts published between January 1 and December 31, 2021. Of these, 202 posts were published during T1 (preimplementation) and 104 posts during T2 (postimplementation). Posts disseminated CFYL oncology nutrition content, including recipes, educational articles, visual graphics, and links to CFYL web content. According to aggregate demographic summaries provided by Meta Business Suite, users who engaged with CFYL content during the 12-month monitoring period were predominantly female (n=9500, 93.2%) and over 35 years of age (n=9311, 91.4%), with the highest engagement present in the group of people aged 55 to 64 years (n=2708, 26.6%). These findings are consistent with broader platform usage patterns for Facebook among midlife adults. The majority of the CFYL audience is based in the United States (n=6708, 71.4%) and identified by the platform as English-language users (n=9419, 93.4%). A smaller proportion of engaged users were identified as Spanish-language users (n=311, 3.1%) with an even lesser number of users residing in a Spanish-speaking country (n=101 from Mexico, 1.1%), indicating that there is also a community of Spanish-speaking individuals residing in the United States who engaged with the CFYL content during this time period (Table 1). Because these demographic data were available only in aggregate platform-provided categories, they describe the engaged audience at the page level rather than post-specific audiences.

**Table 1.** Demographics of users who engaged with Cook for Your Life (CFYL) content during the 12-month monitoring period (N=10,196).

Demographics	n (%)
Gender	
Female	9500 (93.2)
Male	626 (6.1)
Other	70 (0.7)
Location	
United States	6708 (71.4)
Canada	1360 (14.5)
United Kingdom	855 (9.1)
Australia	127 (1.4)
Mexico	102 (1.1)
South Africa	99 (1.1)
Ireland	85 (0.9)
India	53 (0.6)
Age group (y)	
13‐17	4 (0.04)
18‐24	86 (0.1)
25‐34	795 (7.8)
35‐44	1835 (18.0)
45‐54	2486 (24.4)
55‐64	2708 (26.6)
≥65	2282 (22.4)
Language	
English	9419 (93.4)
Spanish	311 (3.1)
French	224 (2.2)
Portuguese	44 (0.4)
Italian	22 (0.2)
Arabic	14 (0.1)
Greek	13 (0.1)
Other	41 (0.4)

### Post Characteristics Associated With Engagement With CFYL Content on Facebook

**Figure 1.**
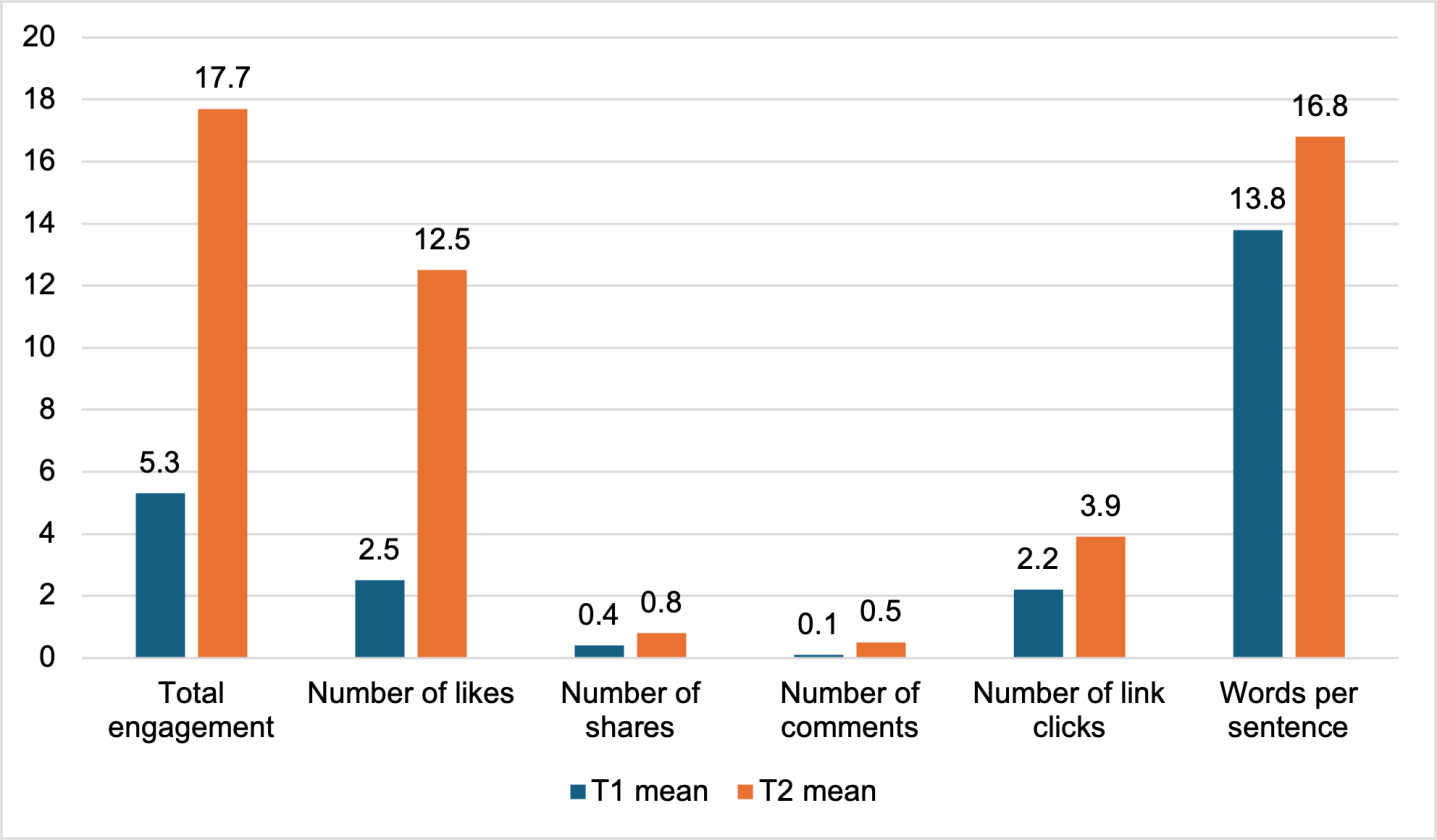
Changes in user engagement with Cook for Your Life (CFYL) across the implementation period.

The overall engagement with posts increased from T1 (mean 5.3, SD 4.2) to T2 (mean 17.7, SD 37.9; *P*<.001; Table 2). Similar increases were observed across engagement components, including likes (T1: mean 2.5, SD 2.0 vs T2: mean 12.5, SD 33.5; *P*<.01), shares (T1: mean 0.4, SD 0.7 vs T2: mean 0.8, SD 1.1; *P*<.01), comments (T1: mean 0.1, SD 0.6 vs T2: mean 0.5, SD 1.3; *P*<.01), and link clicks (T1: mean 2.2, SD 2.6 vs T2: mean 3.9, SD 7.5; *P=*.003; Table 2), underscoring a surge in user interaction with the content. The magnitude of change in user engagement metrics can be visualized in Figure 1. Post format and creative features shifted over time, with a decrease in photography posts (T1: 98.5% vs T2: 67.3%) and increases in text-dominant posts (T1: 1.5% vs T2: 32.7%), infographics (T1: 0.0% vs T2: 7.7%), and branding (T1: 19.8% vs T2: 94.2%; all *P*<.01; Table 2). Similarly, the qualitative content analysis results indicate that the presence of specific content topics within the posts exhibited significant changes during T2. For instance, there were higher proportions of posts focused on cancer (T1: 25.7% vs T2: 58.6%; *P*<.01), nutrition and diet (T1: 64.4% vs T2: 84.6%; *P*<.01), health care support (T1: 18.3% vs T2: 40.4%; *P*<.01), and breast cancer–specific content (T1: 0.5% vs T2: 9.6%; *P*<.01), suggesting a strategic focus on these themes to enhance engagement and promote actionable oncology nutrition information online (Table 2). Bilingual content presentation was also rare in the preimplementation phase but nearly universal after applying the new content strategy (T1: 0.5%, T2: 99.0%; *P*<0.1, Table 2).

**Table 2.** Engagement with posts during 12-month monitoring period by characteristics of posts (N=306).

Engagement type	T1 (n=202)	T2 (n=104)	*P* value
Total engagement, mean (SD)	5.3 (4.2)	17.7 (37.9)	<.001^a^
Number of likes, mean (SD)	2.5 (2.0)	12.5 (33.5)	<.001^a^
Number of shares, mean (SD)	0.4 (0.7)	0.8 (1.1)	<.001^a^
Number of comments, mean (SD)	0.1 (0.6)	0.5 (1.3)	<.001^a^
Number of link clicks, mean (SD)	2.2 (2.6)	3.9 (7.5)	.003^a^
Words per sentence, mean (SD)	13.8 (3.8)	16.8 (3.8)	<.001^a^
Image content, n (%)			
Text	3 (1.5)	34 (32.7)	<.001^a^
Photography	199 (98.5)	70 (67.3)	<.001^a^
Infographic	0 (0.0)	8 (7.7)	<.001^a^
GIF	0 (0.0)	3 (2.9)	.02^a^
Branding	40 (19.8)	98 (94.2)	<.001^a^
Square shape	12 (5.9)	94 (90.4)	<.001^a^
Rectangular shape	190 (94.1)	10 (9.6)	<.001^a^
Bilingual post	1 (0.5)	103 (99.0)	<.001^a^
Content topic, n (%)			
Immune system	3 (1.5)	7 (6.7)	.01^a^
Chemotherapy	1 (1.5)	4 (3.9)	.03^a^
Radiation	1 (0.5)	2 (1.9)	.23
Cancer	52 (25.7)	61 (58.6)	<.001^a^
Survivorship	4 (2.0)	1 (1.0)	.50
Caregiver	0 (0.0)	2 (1.9)	.05^a^
Health care support	37 (18.3)	42 (40.4)	<.001^a^
Prevention	41 (20.3)	30 (28.8)	.09
Nutrition and diet	130 (64.4)	88 (84.6)	<.001^a^
Heart health	0 (0.0)	1 (1.0)	.16
Cancer type			
Gynecological	1 (0.5)	0 (0.0)	.47
Colorectal	1 (0.5)	1 (1.0)	.63
Breast	1 (0.5)	10 (9.6)	<.001^a^
Blood	0 (0.0)	2 (1.9)	.048^a^
Bladder	1 (0.5)	0 (0.0)	.47

a Indicates statistical significance at *P*<.05.

### Engagement by Lexical Content

The results from the natural language processing analysis using LIWC produced a large number of linguistic and semantic dimensions, which may be interpreted as an exploratory descriptive comparison of language style across time periods before and after the implementation of the targeted social media content strategy. T2 posts showed higher Analytic scores, longer sentences, and lower rates of affect- and cognition-related terms, indicating a shift toward more formal, informational language. The average number of words per sentence increased from mean 13.83 (SD 3.84) at T1 to mean 16.81 (SD 3.85) at T2 (*P*<.01; Table 2), demonstrating a shift toward longer sentence structures. The linguistic analysis revealed that posts in the latter time period (T2) exhibited a substantial increase in the use of “analytical” language from mean 80.04 (SD 18.05) at T1 to mean 87.70 (SD 5.66) at T2 (*P*<.001; Table 3), along with greater use of “Big Words” from mean 32.85 (SD 7.48) at T1 to mean 39.66 (SD 5.27) at T2 (*P*<.01; Table 3), suggesting a shift toward evidence-based logic and the use of complex medical terminology related to health science, oncology, and nutrition. Conversely, the use of words included in the LIWC dictionary declined from mean 64.92 (SD 8.34) at T1 to mean 37.23 (SD 5.76) at T2 (*P*<.001; Table 3), suggesting the use of a scientific vocabulary with uncommon words appearing more frequently in the text of T2 posts. Other linguistic style indices such as Linguistic (mean 44.84, SD 8.49 to mean 19.10, SD 3.49; *P*<.001) and Function words (mean 31.18, SD 8.48 to mean 12.36, SD 3.02; *P*<.01) also declined from T1 to T2 (Table 3).

**Table 3.** Lexical content by time period (N=306 posts).

Lexical category	T1, mean (SD)	T2, mean (SD)	*P* value
Analytical thinking	80.04 (18.05)	87.70 (5.66)	<.001
Dictionary	64.92 (8.34)	37.23 (5.76)	<.001
Linguistic	44.84 (8.49)	19.10 (3.49)	<.001
Tone	39.95 (34.60)	28.86 (19.79)	.003
Big words	32.85 (7.48)	39.66 (5.27)	<.001
Function	31.18 (8.48)	12.36 (3.02)	<.001
Authentic	19.03 (21.64)	1.12 (0.60)	<.001
Drives			
Affiliation	1.77 (2.41)	1.12 (1.20)	.009
Cognition			
Cognition	7.33 (4.6)	3.0 (1.8)	<.001
Cognitive processes	6.65 (4.47)	2.64 (1.59)	<.001
Differentiation	3.08 (2.70)	0.85 (0.80)	<.001
Tentative	2.38 (2.22)	0.50 (0.61)	<.001
Insight	1.01 (1.92)	0.60 (0.75)	.02
Discrepancy	1.00 (1.48)	0.49 (0.76)	.001
Certitude	0.43 (1.00)	0.08 (0.25)	<.001
Affect			
Affect	6.11 (3.83)	3.76 (1.50)	<.001
Positive tone	3.59 (3.25)	2.07 (1.25)	<.001
Emotion	2.88 (2.08)	1.62 (0.93)	<.001
Negative tone	2.44 (1.54)	1.65 (0.83)	<.001
Negative emotion	2.15 (1.26)	1.34 (0.64)	<.001
Positive emotion	0.59 (1.38)	0.24 (0.42)	.01
Social processes			
Social processes	5.96 (4.05)	4.17 (2.54)	<.001
Social referents	4.58 (3.41)	2.66 (1.50)	<.001
Family	0.05 (0.35)	0.24 (0.42)	<.001
Conflict	0.04 (0.36)	0.14 (0.27)	.01
Male references	0.01 (0.14)	0.23 (0.43)	<.001
Culture			
Culture	2.23 (1.37)	1.45 (0.67)	<.001
Technology	2.06 (1.23)	1.31 (0.63)	<.001
Politics	0.01 (0.12)	0.07 (0.22)	.001
Physical			
Health	1.96 (3.57)	6.69 (3.29)	<.001
Food	6.57 (4.44)	3.81 (2.39)	<.001
Illness	0.83 (2.08)	5.64 (3.09)	<.001
States			
Acquire	1.12 (1.52)	0.37 (0.51)	<.001
Motives			
Allure	4.74 (3.48)	1.87 (1.10)	<.001
Perception			
Physical	8.63 (5.33)	10.84 (3.57)	<.001
Perception	7.26 (4.30)	2.09 (1.46)	<.001
Space	4.87 (3.15)	1.06 (0.97)	<.001
Present focus	2.85 (2.42)	1.08 (0.88)	<.001
Time	2.64 (2.81)	1.06 (1.03)	<.001
Conversational	2.18 (1.36)	1.47 (0.69)	<.001
Netspeak	2.14 (1.33)	1.45 (0.68)	<.001
Future focus	0.82 (1.59)	0.31 (0.48)	.001
Motion	0.71 (1.36)	0.19 (0.34)	<.001
Visual	0.63 (1.34)	0.34 (0.58)	.04
Feeling	0.59 (1.34)	0.19 (0.42)	.003
Fulfill	0.44 (1.09)	0.14 (0.34)	.005
Want	0.27 (0.80)	0.07 (0.31)	.02

Informal language markers such as the use of conversational language (mean 2.18, SD 1.36 to mean 1.47, SD 0.69; *P*<.001) and netspeak (mean 2.14, SD 1.33 to mean 1.45, SD 0.68; *P*<.001) decreased significantly from T1 to T2 (, Table 3), along with a decrease in the Authentic summary score (mean 19.03, SD 21.64 to mean 1.12, SD 0.60; *P*<.001; Table 3). Affect-related categories revealed significant changes in emotional tone, including affect (mean 6.11, SD 3.83 to mean 3.76, SD 1.50; *P*<.001), positive emotion (mean 0.59, SD 1.38 to mean 0.24, SD 0.42; *P*=.01), and negative emotion (mean 2.15, SD 1.26 to mean 1.34, SD 0.64; *P*<.001), with decreases in both positive tone (mean 3.59, SD 3.25 to mean 2.07, SD 1.25; *P*<.001) and negative tone (mean 2.44, SD 1.54 to mean 1.65, SD 0.83; *P*<.001; Table 3). These findings signify a shift toward less emotionally charged language in T2 posts.

Cognition-related categories were lower at T2, including cognitive processes (mean 6.65, SD 4.47 to mean 2.64, SD 1.59; *P*<.001), tentative language (mean 2.38, SD 2.22 to mean 0.50, SD 0.61; *P*<.001), discrepancy (mean 1.00, SD 1.48 to mean 0.49, SD 0.76; *P*<.001), and certitude (mean 0.43, SD 1.00 to mean 0.08, SD 0.25; *P*<.001; Table 3), suggesting a shift toward certainty and scientific reasoning. There was also less perception-related language used in T2 posts, indicating that the content shifted away from describing subjective experiences with sensory terms and toward promoting objective scientific evidence (mean 7.26, SD 4.30 to mean 2.09, SD 1.46; *P*<.001). Additionally, the use of physical language related to health (mean 1.96, SD 3.57 to mean 6.69, SD 3.29; *P*<.001) and illness terms (mean 0.83, SD 2.08 to mean 5.64, SD 3.09; *P*<.001) was greater at T2, suggesting a shift in content topics toward cancer-centered language.

Several characteristics of posts were associated with engagement in univariate regression analyses controlling for the time period (Table 4). First, posts that used the bilingual multiformat content strategy were associated with higher engagement (OR 1.72, 95% CI 1.05‐2.80; *P*=.03). The presence of CFYL branding (OR 1.08, 95% CI 1.04‐1.13; *P*<.001) and square-formatted images (OR 1.11, 95% CI 1.06‐1.17; *P*<.001) was also associated with higher engagement, whereas rectangular-formatted posts were associated with lower engagement (OR 0.90, 95% CI 0.86‐0.94; *P*<.001; Table 4). Only bilingual content was associated with increased odds of engagement after adjusting for the time point (data not shown). These findings underscore the influence of linguistic and thematic elements on user engagement with CFYL content on Facebook, even when adjusting for variations in the time period of the posts.

**Table 4.** Characteristics of posts and lexical content associated with engagement across all posts controlling for time period (N=306).

Post characteristics	OR (95% CI)	*P* value
Post structure		
Mostly text	1.00 (0.99‐1.01)	.61
Mostly image	1.00 (0.98-1.01)	.61
Infographic	0.99 (0.93‐1.06)	.78
With branding	1.08 (1.04‐1.13)	<.001^a^
GIF	1.00 (0.97‐1.04)	.80
Square shape	1.11 (1.06‐1.17)	<.001^a^
Rectangular shape	0.90 (0.86‐0.94)	<.001^a^
Bilingual	1.72 (1.05‐2.80)	.03^a^
Topic		
Immune system	0.99 (0.95‐1.04)	.83
Chemotherapy	0.97 (0.84‐1.13)	.74
Radiation	0.97 (0.79‐1.19)	.77
Cancer	1.00 (0.99‐1.01)	.33
Survivorship	0.98 (0.96‐1.12)	.77

a Indicates statistical significance at *P*<.05.

Changes observed in engagement metrics during T2 coincided with co-occurring shifts in post design and language. Specifically, the increase in branded and square-formatted creative (manual coding; Table 2) aligned with positive associations between these features and engagement in regression analyses (Table 4). Similarly, increases in posts coded as cancer, health care support, and nutrition-focused (Table 2) aligned with LIWC shifts showing more health and illness language and less food language (Table 3), suggesting that posts increasingly framed nutrition within cancer and treatment contexts during the strategy period.

## Discussion

### Principal Findings

This study analyzed 306 original CFYL-authored Facebook posts published over 12 months and described both overall engagement volume and postlevel engagement patterns before and after the implementation of a bilingual, multiformat content strategy. Across the observation window, engagement with CFYL content was concentrated among women, adults aged 35 years and older, and predominantly English-language users based in the United States, according to aggregate platform demographic summaries. These demographic results are in contrast to the epidemiology of cancer in America, where there is a much broader distribution of demographics across patients and survivors. One reason for this finding may be that younger adults are more likely to engage with social media platforms and have demonstrated high rates of digital health literacy. This distinct cohort could also be interpreted as the population of female caregivers who provide the invisible labor of seeking and implementing health information within their home and community. The demographic data might suggest that women act as the primary drivers of health-related caregiving and nutrition management within their social support networks.

In analyses controlling for the time period, bilingual posts had higher odds of engagement, and creative features, including CFYL branding and formatted images, were also positively associated with engagement. Linguistically, posts produced with this content strategy used more analytical language and longer sentences, with fewer conversational, authentic, affective, and cognitive terms in T2 than T1. These results amount to more formal, scientific language and less emotionally charged vocabulary used in T2, aligning with best practices for organizational evidence-based messaging. Content-related lexical categories also shifted, with increases in health and illness terms, indicating a greater emphasis on nutrition in the context of cancer and treatment-related concerns. Metrics of engagements per post increased over time, and in models that adjusted for the time period, bilingual posts had higher odds of engagement.

Creative features such as institutional branding and purpose-designed visual content were also positively associated with engagement. Visual branding implementation associated with CFYL and the Fred Hutch Cancer Center may have the potential to drive higher credibility among the target content audience. Given the observational design and co-occurring changes to content and format, these results should be interpreted as associations rather than causal effects. However, these findings emphasize the importance of strategic social media approaches in increasing user interaction with high-quality cancer information on social media platforms [32-34].

### Comparison to Prior Work

Widespread access to the internet has drastically shifted the sources of health information from health care providers to user-generated online content. Simultaneously, access to high-quality cancer information online may not be equitable. This is especially true in a global online community where the source and credibility of cancer information are not always transparent. Incorporating bilingual cancer content on the CFYL Facebook page extended accessibility for Spanish-speaking individuals. Bilingual social media strategies have been documented to promote user engagement with online health promotion content in other settings as well, with Latino Facebook users tending to show more engagement with either Spanish or bilingual posts compared to English posts [35]. This suggests that cancer centers, hospitals, and cancer advocacy groups that intend to promote accessible cancer information online should strongly consider a bilingual approach, matching the linguistic needs of the target population.

Differences in health literacy on a global scale also emphasize the need for cancer information to be delivered at an appropriate literacy level, particularly given our current digital age where people worldwide have access to social media content [36]. The observed rise in the use of complex language elements represents a conscious effort to make the content more informative and scientifically accurate. However, the longer sentence structure and use of more complex, analytical language may present comprehension challenges for diverse audiences, such as individuals with varying levels of health literacy. Simplifying language in health communication is a recognized strategy to ensure that information is easily understood and accessible to a wider spectrum of readers. It may be valuable for CFYL to assess the health literacy of their audience and tailor social media content in order to make critical information about cancer nutrition more accessible and understandable to a broader public.

Moreover, the shift toward less emotionally charged language, as evidenced by the decrease in language that uses both positive and negative tones, aligns with the expected findings observed in organizational communication. Previous research has indicated that organizations, especially those in the health domain, tend to use fewer emotionally charged words compared to individual users [37]. This strategic approach aims to maintain a professional and informative tone, avoiding potential misinterpretations that emotionally charged language might introduce. It is also plausible that less emotional, more objective language is interpreted as being more credible and scientifically accurate. We also observed declines in perceptual processes (see/hear/feel), which likely reflect fewer sensory descriptors and a more didactic, informational tone used in the new content strategy. The decline of conversational and internet-specific language suggests fewer informal linguistic markers, which aligns with the educational content strategy used by cancer research institutes. This can increase credibility for an audience seeking trustworthy nutrition guidance.

We found that visual content such as photo media, branding, and infographics are useful tools to improve engagement with cancer nutrition information on social media, which is supported by prior literature on the topic within the field of health information and cancer research [32-34]. Visual content is commonly used across social media platforms, and social media posts with visual content are more frequently shared with other users and are associated with engagement metrics (including likes and comments) [34]. In addition to its impact on user engagement, visual content improves persuasive impact [38]. Other factors associated with higher engagement across time points were branding, square-shaped posts, and bilingual content. Square-shaped posts represent purposively selected photos that were identified by the CFYL content creators as opposed to rectangular-shaped posts, which represent preselected images that are input automatically when adding an external link into a Facebook post. Therefore, this finding does not imply an attraction to a specific geometrical shape but it suggests a preference for intentional photos over simple links. Institutions and researchers aiming to improve engagement with their social media content may use these strategies.

### Strengths and Limitations

Strengths of this study design include the integration of platform analytics with manual content coding and the addition of linguistic analysis to characterize message features. There are also limitations of this approach that should be considered in the interpretation of our results. First, we cannot attribute engagement changes solely to bilingual content due to the multiple changes that occurred around the same time as the bilingual strategy (eg, more branding, more square images, and topic shifts). Because exposure varied and multiple changes occurred alongside the strategy, causality cannot be inferred. We attempted to mitigate this by comparing post characteristics across periods and by including the time period in regression models; however, residual confounding by unmeasured time-varying factors likely remains. Future studies should use longer, balanced observation windows, staggered rollouts, or interrupted time-series designs to strengthen causal inference.

Second, we could not directly compare engagement by language because nearly all posts after implementation were bilingual. This limits inferences about the relative contribution of Spanish vs English content. Although we examined overall engagement changes and the presence of bilingual formatting, this approach cannot isolate language-specific effects. Future work could stratify analyses by audience language segments when platform analytics allow it. Another factor to consider is that engagement counts are influenced by audience exposure (reach and impressions), which can vary by post and over time. Our primary analyses did not explicitly model exposure as an offset to normalize engagement rates for delivery. As a result, posts with wider distribution of engagement may appear to perform better independent of their content.

Third, there are threats to the generalizability of these findings in other settings and online communities. The data reflect a single oncology nutrition platform and an engaged audience that was predominantly female and largely based in the United States, although demographic granularity was limited to platform-provided aggregates. These findings are limited to Facebook, and characteristics may not represent other populations or platforms with distinct content formats and algorithms. Replication across additional cancer centers, community organizations, and other social networks (eg, Instagram, YouTube, and TikTok) would strengthen the external validity of these findings.

We acknowledge that the asymmetric time windows to assess preimplementation and postimplementation of this content strategy may also introduce seasonality and exposure differences into our results. Additionally, changing temporal, cultural, and algorithmic dynamics may have altered engagement patterns independent of content during the observation period. We partially addressed this by incorporating time period comparisons, but more granular controls (eg, calendar month, day of the week, or platform policy change markers) would better account for temporal and algorithmic shifts. Extending monitoring across multiple years in future studies may also help separate strategy effects from seasonality.

### Future Directions

In the future, novel digital media and technological tools (eg, eye-tracking and web-monitoring) may be useful for determining how individuals evaluate the quality of cancer nutrition information and the features that promote online engagement with cancer nutrition information. This technology has been previously implemented in research to record users’ viewing patterns on web-delivered information and provides a physiological measure linked to cognitive processing that may indicate the presence of active or passive engagement [39,40]. Data from eye-tracking can assess how users process online information such as social media content [41,42]. In the context of public health research, eye-tracking technology improves the understanding of how online health promotion content attracts attention from users [39,41,43].

The association between social media use and oncology nutrition knowledge is another topic that should be explored further, particularly because engaged social media users are more likely to be aware of other prevention measures, such as cancer screening [44,45]. Similarly, improving engagement with oncology nutrition content on social media may promote healthy dietary choices for cancer prevention and patients with cancer. The dietary needs of patients with cancer undergoing treatment such as chemotherapy are different from those of cancer survivors or caregivers aiming to reduce cancer risk. Therefore, oncology nutrition interventions through social media may benefit from addressing these individualized needs through targeted social media content. Importantly, future work should assess how exposure to online cancer information influences users’ health beliefs, perceived cancer risk, trust in nutrition recommendations, and subsequent health behaviors.

It is also relevant to examine how these findings translate to other social media platforms with distinct audiences, content formats, and engagement algorithms such as Instagram, TikTok, and Snapchat. Each of these platforms reaches different demographic groups and prioritizes different types of content, particularly short-form video, visual storytelling, and creator-driven posts. Studying oncology nutrition messaging across these environments may help determine whether bilingual strategies, visual elements, or linguistic elements operate similarly on platforms that emphasize rapid, algorithm-driven content exposure. Understanding these mechanisms will be essential for identifying which platform features most effectively support evidence-based cancer prevention and nutrition education and for ensuring that such content reaches diverse communities that may not engage with Facebook.

Going forward, providers and staff can leverage cancer nutrition websites and social media to guide patients to high-quality cancer information by incorporating visual content such as photos, branding, and infographics, which improve social engagement, as well as including more analytical language and a neutral emotional tone within a multilanguage content strategy. It is important for providers to be aware of disparities in accessing information and to make cancer information available in different languages and at varying literacy levels. Another area with clinical relevance is to consider the specific dietary needs of patients with cancer undergoing treatment and provide targeted social media content to address these dietary needs both during treatment and across the cancer control continuum.

### Conclusion

This study describes how CFYL, an online cancer nutrition community, disseminates information about nutrition education and cancer prevention. The results of this analysis demonstrate that implementing a dedicated bilingual, multiformat social media content strategy is associated with higher audience engagement with oncology nutrition posts. We describe strategies to increase engagement with online social media content disseminating nutrition-related cancer prevention and survivorship information. In models adjusted for the time period, posts that were bilingual, branded, and formatted performed better, and the language profile shifted toward more analytical, emotionally neutral text.

These associations were observed alongside concurrent changes in creative elements and content delivery, and exposure varied across posts. As such, causal inferences cannot be made. Even so, these findings suggest practical signals for designing bilingual, branded, multiformat content to support the equitable dissemination of evidence-based cancer nutrition information. Studying online language use may help cancer organizations strengthen the rationale for investing in bilingual social media cancer communication initiatives tailored to the language needs of the communities they serve. Future work should test language presentation more directly and evaluate how exposure to such content influences health beliefs and downstream behaviors across multiple social media platforms. Overall, these findings provide an initial context for communicating about nutrition with patients with cancer, survivors, and caregivers on Facebook and other social media platforms. These results are a step toward implementing more supportive interventions for the quality dissemination of cancer nutrition information on social media.

## Supplementary material

10.2196/69886 Multimedia Appendix 1 Qualitative codebook.

## Abbreviations

CFYL: Cook For Your Life
FHCC: Fred Hutchinson Cancer Center
LIWC: Linguistic Inquiry and Word Count
LOL: laughing out loud
OMG: oh my god
OR: odds ratio
