# Supplementary material for: Understanding Social Media Strategies to Improve Engagement in and Equitable Dissemination of Online Cancer Nutrition Information: Cross-Sectional Study
Source: JMIR Cancer. 2026 Aug 14;12:e69886. doi: 10.2196/69886 (PMC13475934; doi:10.2196/69886)
Supplement: Supplementary file 1 [file cancer-v12-e69886-s001.docx]

**Multimedia Appendix 1: Qualitative Codebook**

| **Image Content** | **Content Topic** | **Cancer type** |
| --- | --- | --- |
| Text | Immune system | Gynecological |
| Photography | Chemotherapy | Colorectal |
| Infographic | Radiation | Breast |
| GIF | Cancer | Blood |
| Branding | Survivorship | Bladder |
| Square shape | Caregiver |  |
| Rectangular shape | Healthcare Support |  |
| Bilingual post | Prevention |  |
|  | Nutrition & diet |  |
|  | Heart Health |  |
